# High proportions of asymptomatic and submicroscopic *Plasmodium vivax* infections in a peri-urban area of low transmission in the Brazilian Amazon

**DOI:** 10.1186/s13071-018-2787-7

**Published:** 2018-03-20

**Authors:** Anne C. G. Almeida, Andrea Kuehn, Arthur J. M. Castro, Sheila Vitor-Silva, Erick F. G. Figueiredo, Larissa W. Brasil, Marcelo A. M. Brito, Vanderson S. Sampaio, Quique Bassat, Ingrid Felger, Wanderli P. Tadei, Wuelton M. Monteiro, Ivo Mueller, Marcus V. G. Lacerda

**Affiliations:** 1 Fundação de Medicina Tropical Dr. Heitor Vieira Dourado (FMT-HVD), Av. Pedro Teixeira, N.25, Dom Pedro, Manaus, Amazonas CEP: 69040-000 Brazil; 2 Universidade do Estado do Amazonas (UEA), Av. Djalma Batista, N. 3578, Flores, Manaus, Amazonas CEP: 69005-010 Brazil; 3 ISGlobal, Barcelona Ctr. Int. Health Res. (CRESIB), Hospital Clínic - Universitat de Barcelona, Carrer del Rosselló, 132, 08036 Barcelona, Spain; 4 Instituto Nacional de Pesquisas da Amazônia (INPA), Av. André Araújo, N. 2.936, Petrópolis, Manaus, CEP: 69067-375 Brazil; 5 Fundação de Vigilância em Saúde do Amazonas, Sala de Análise de Situação em Saúde, Av. Torquato Tapajós, N. 6132, Colônia Santo Antonio, Manaus, CEP:69093-018 Brazil; 6 Centro de Investigação em Saúde de Manhiça (CISM), Rua 12, Cambeve, Vila de Manhiça, CP 1929 Maputo, Mozambique; 7 Institució Catalana de Recerca i Estudis Avançats (ICREA), Passeig Lluís Companys, 23 08010 Barcelona, Spain; 8 Swiss Tropical and Public Health Institute, Socinstrasse 57, 4051 Basel, Switzerland; 9 University of Basel, Petersplatz 1, 4001 Basel, Switzerland; 10 Walter and Eliza Hall Institute, Parkville, Australia; 11 Instituto de Pesquisas Leônidas e Maria Deane (ILMD), Manaus, Brazil

**Keywords:** Malaria, *Plasmodium vivax*, Asymptomatic infection, Submicroscopic infection, Gametocyte carriage

## Abstract

**Background:**

Population-based studies conducted in Latin America have shown a high proportion of asymptomatic and submicroscopic malarial infections. Considering efforts aiming at regional elimination, it is important to investigate the role of this asymptomatic reservoir in malaria transmission in peri-urban areas. This study aimed to estimate the prevalence of *Plasmodium* spp. and gametocyte burden on symptomatic and asymptomatic infections in the Brazilian Amazon.

**Results:**

Two cross-sectional household surveys (CS) were conducted including all inhabitants in a peri-urban area of Manaus, western Amazonas State, Brazil. Malaria parasites were detected by light microscopy (LM) and qPCR. Sexual stages of *Plasmodium* spp. were detected by LM and RT-qPCR. A total of 4083 participants were enrolled during the two surveys. In CS1, the prevalence of *Plasmodium vivax* infections was 4.3% (86/2010) by qPCR and 1.6% (32/2010) by LM. Fifty percent (43/86) of *P. vivax* infected individuals (qPCR) carried *P. vivax* gametocytes. In CS2, 3.4% (70/2073) of participants had qPCR-detectable *P. vivax* infections, of which 42.9% (30/70) of infections were gametocyte positive. The *P. vivax* parasite density was associated with gametocyte carriage (*P* < 0.001). Sixty-seven percent of *P. vivax* infected individuals and 53.4% of *P. vivax* gametocyte carriers were asymptomatic.

**Conclusions:**

This study confirms a substantial proportion of asymptomatic and submicroscopic *P. vivax* infections in the study area. Most asymptomatic individuals carried gametocytes and presented low asexual parasitemia. This reservoir actively contributes to malaria transmission in the Brazilian Amazon, underscoring a need to implement more efficient control and elimination strategies.

**Electronic supplementary material:**

The online version of this article (10.1186/s13071-018-2787-7) contains supplementary material, which is available to authorized users.

## Background

The past two decades have witnessed impressive success in the reduction of malaria incidence in Latin America, with the number of confirmed cases decreasing by 31.0% from 2000 to 2015. Nevertheless, malaria remains a major public health concern in endemic countries, with approximately 90 million people being at risk of infection and an estimated 216 million cases worldwide in 2016 [1]. In 2013 more than 40% of malaria cases in Latin America occurred in Brazil and, within that country, more than 99% of all cases were concentrated in the Amazon region [2]. From 2005 to 2015, malaria incidence in Brazil decreased considerably, as evidenced by reductions in Annual Parasite Index (79.2%) and total number of cases (76.5%). This trend was accompanied by an even more pronounced decline in the proportion of *Plasmodium falciparum*, with *Plasmodium vivax* predominance progressively increasing to peak at 88.4% of total malaria cases in 2015 [3]. Such an impressive reduction of cases is likely attributable to the governmental program focusing on early case detection and treatment [4]. Despite the relative success of control efforts, malaria transmission still occurs in the Amazon region with major outbreaks recorded in 1999 and 2005 that may have been in part due to deforestation, mining activities and new unplanned rural settlements resulting in environmental changes favouring malaria transmission [2, 5]. After the 1970s, a large migratory influx from the rural zones to the peripheries of bigger cities in the Amazon basin resulted in a precarious and uncontrolled urbanization process, increasing human exposure to anopheline vectors in peri-urban areas [6, 7]. In Brazilian Amazon, *Anopheles darlingi* is the main malaria vector [8, 9].

Latin America is considered a low to moderate malaria transmission region. Recent population based surveys reported prevalence rates below 6.0% by LM [10–14]. PCR analyses revealed higher prevalences, ranging between 5.0–17.0% [10–14]. In some studies the majority of infected individuals did not show malaria-related symptoms [12, 15]. These results suggest that a large proportion of infections was submicroscopic and asymptomatic and would not be detected by routine diagnostic methods, although asymptomatic infections are believed to substantially contribute to malaria transmission. Thus, in this renewed elimination era, it may seem appropriate to reconsider the role of the asymptomatic reservoir as a likely source of transmission, especially in low transmission settings. Specifically and actively targeting this reservoir, rather than relying on passive detection of clinically symptomatic malaria infections, may be essential to achieve local elimination.

Most studies examining prevalence of *Plasmodium* spp. in the Amazon region were carried out in small rural or riverine settlements. Additionally, very few studies assessed the prevalence and density of gametocytes, the parasite stage responsible for transmission from human to mosquito vectors. This study aimed to determine the prevalence of *Plasmodium* spp. and the gametocyte burden in symptomatic and asymptomatic infections in a peri-urban area in the Brazilian Amazon.

## Methods

In order to estimate the reservoir of *Plasmodium* spp. infected individuals contributing to malaria transmission, we conducted two large-scale cross-sectional surveys in a peri-urban area of Manaus, located in the western part of the Amazonas State, Brazil. The presence of sexual parasite stages in both symptomatic and asymptomatic infected individuals was also investigated.

### Study site

The municipality of Manaus is located on the river Rio Negro banks, in the northeast of the Amazonas State. The municipality has an estimated 2,094,301 inhabitants of whom the great majority resides in urban and peri-urban areas [16]. Intensive migration, as well as persisting challenges to maintaining an adequate epidemiological and entomological surveillance, are contributing to the actively ongoing sustained malaria transmission in the region. Risk of infection varies between low and high risk in rural and peri-urban areas. Highest incidence of malaria cases is commonly observed from May to September, in the dry season when water levels are declining [17].

**Fig. 1:**
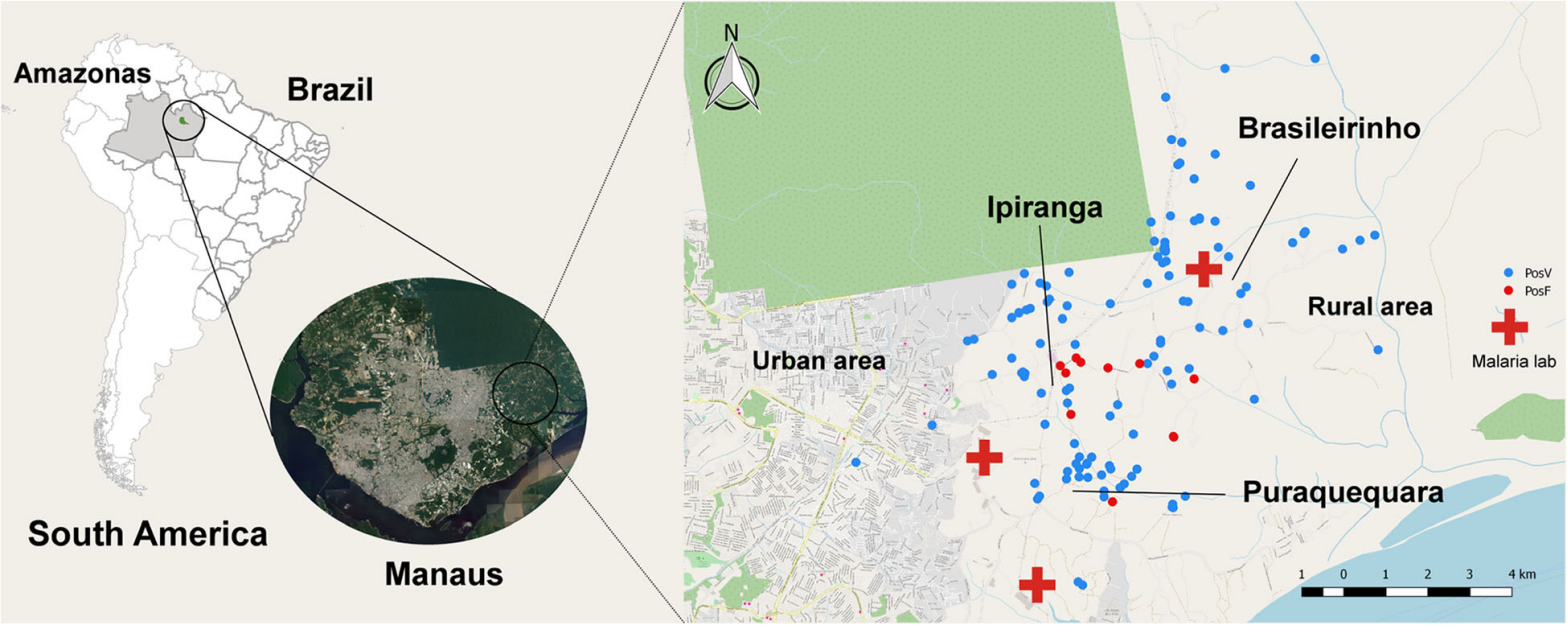
Study area and distribution of malaria infected individuals (red: *P. falciparum*, blue: *P. vivax*) enrolled at cross-sectional surveys, detected by qPCR

The three communities in which this study was conducted, Brasileirinho, Ipiranga and Puraquequara, are located in the peri-urban area of Manaus (Fig. 1). The main occupation is in the agricultural sector including pisciculture. Many people travel to the city daily for work. There are many places for religious conventions and leisure activities, where people from the city stay for the weekend and public holidays. Malaria infections among these visitors from non-endemic areas of Manaus are common. Sanitation and garbage collection services are not available, water is collected from natural springs or rivers as well as received from a city service. According to a census performed by a field team of Fundação de Medicina Tropical Dr. Heitor Vieira Dourado (FMT-HVD) the population of the study area (Brasileirinho, Ipiranga and the northern part of Puraquequara, adjacent to Brasileirinho) was estimated at around 2400 inhabitants in 2012. Each community has access to one malaria clinic specifically conducting malaria diagnosis by microscopy and treatment of cases. Health agents based in this clinic regularly visit homes in order to ensure early detection and treatment of cases. Sporadically they perform active detection of febrile cases in the neighbourhood of confirmed cases.

### Study population and survey method

Two cross-sectional surveys were conducted on a household-basis, including all inhabitants of all ages of the study area that were willing to participate in the study. In case of absence of any family members, the field team returned to the respective house for a second time. For each study participant a questionnaire was completed, containing personal information (age, gender, occupation, pregnancy, history of travel), information on malaria preventive measures, previous malaria episodes and current health status. The first cross-sectional survey (CS1) was conducted from mid-November 2012 to beginning of January 2013, at the beginning of the rainy season, and the second one (CS2) from the beginning of August 2013 to mid-September 2013, at the end of the dry season.

### Sample collection

Upon participant enrolment, a 300 μl blood sample was collected from each patient *via* finger puncture using Microtainer® tubes containing EDTA and sodium fluoride (Becton Dickinson, NJ, USA). In infants, blood was obtained by puncture of the heel or toe. Within 1 h of collection, 50 μl of blood was transferred into a reaction tube containing 250 μl of RNAprotect® (QIAGEN, Hilden, Germany) in order to preserve RNA for downstream analyses [18] and 200 μl of whole blood were transferred to another reaction tube. Samples were maintained in cooling boxes until arrival in the laboratory, where the 200 μl sample was separated into red blood cell (RBC) pellet and plasma. All samples were frozen at -80 °C until further processing. If the collected blood volume was< 250 μl, the actual volume was recorded.

In order to assess the submicroscopic proportion of infections, during CS1 two thick blood smears were prepared for all participants. One slide was analysed at the local malaria outpost (for diagnostics purposes only) and the second was analysed by a microscopist at FMT-HVD for confirmation of species and parasitemia. In cases of a positive result, participants were treated by health post staff within 24 h according to the national guidelines of the Ministry of Health [19]. During CS2 only in the case of symptoms related to malaria a thick blood smear was prepared for diagnosis according to the national guidelines. Participants with positive result received prompt treatment.

### Classification of infection status

All participants were tested for *P. vivax* and *P. falciparum* by qPCR (and in CS1 additionally by LM) to determine positivity of infection. Individuals with body temperature above 37.5 °C at blood collection or in the past 48 h were considered as febrile. An asymptomatic infection was defined as the presence of a malarial infection in the absence of fever and any other malaria related symptoms (chills, sweating, headache, vomit, abdominal pain) at the moment of sample collection, or anytime during the preceding 48 h. Individuals developing symptoms within 30 days after blood collection (data obtained from SIVEP-Malaria database, Information System for Epidemiological Surveillance of Malaria of the Brazilian Ministry of Health) were also considered symptomatic and therefore excluded from the asymptomatic group.

### Detection of *Plasmodium* spp. parasites by light microscopy

Thick blood smears were Giemsa-stained according to World Health Organization guidelines [20]. Numbers of asexual blood stage parasites and gametocytes were determined per 200 leukocytes and parasites per μl were calculated using an assumed density of 6000 leukocytes per μl [21]. For quality control purposes, all slides with positive results and 10% of all negative slides were read by a second microscopist. In case of a positive/negative discrepancy, or when parasitemias were in disagreement by more than 25.0%, the slides were re-read by a third microscopist and only the two most similar results were used for calculation of final parasite density. All PCR positive samples were examined at least twice by LM.

### DNA and RNA extraction

Pelleted RBCs obtained from 200 μl blood were resuspended in PBS and genomic DNA was extracted using FavorPrep^TM^ 96-well Genomic DNA Kit (Favorgen, Ping-Tung, Taiwan) according to the manufacturer’s instructions. DNA was eluted with 2 × 100 μl of elution buffer and stored at -20 °C until analysed by PCR. If the amount of whole blood available for DNA extraction was ≤ 100 μl, the DNA volume was vacuum concentrated (Concentrator 5301, Eppendorf, Hamburg, Germany) according to the volume originally collected. RNA from whole blood stored in RNAprotect was extracted by means of the RNeasy® Plus 96 kit (Qiagen, Hilden, Germany) as described elsewhere [18].

### Detection of *Plasmodium* spp. by qPCR and detection of gametocyte-specific transcripts by RT-qPCR

All DNA samples were subjected to QMAL Taqman qPCR to detect *Plasmodium* spp. by targeting a conserved region of the *18S* rRNA gene [18]. QMAL-positive samples were further analysed by Taqman qPCR assays detecting species-specific sequences of *18S* rRNA gene of *P. falciparum* and *P. vivax*, as previously described [18, 22]. For detection of *P. falciparum* a modified reverse primer was used (Additional file 1: Table S1) [23]. For quantification of *18S* rRNA gene copy numbers, in each experiment three dilutions of plasmids containing the respective region targeted were included in triplicates (10^2^, 10^4^ and 10^6^ copies/μl). All samples positive for *P. vivax* and/or *P. falciparum* were included in RT-qPCR assays detecting the respective transcripts of *p25* gene (*pvs25* in *P. vivax* and *pfs25* in *P. falciparum*), which is specifically expressed in gametocytes. For quantification of *pvs25* and *pfs25* transcripts, standard plasmids containing the region targeted by the RT-qPCR were included in each run. All qPCR and RT-qPCR assays were ran in the 7500 Fast Real-Time PCR System (Applied Biosystems, California, USA). Primer and probe sequences, composition of reaction mixes, PCR profiles and the detection limit for each assay are listed in Additional file 1: Table S1, Additional file 2: Table S2 and Additional file 3: Table S3.

### Statistical analyses

Questionnaire data were imported into databases using Cardiff TeleForm version 10.4.1 (Cardiff Software). Individual databases were combined in Microsoft Access 2010. Statistical analyses were performed with STATA® 13 (StataCorp LP, College Station, TX, USA). Differences in participant characteristics, parasite prevalence and carriage of gametocyte between the two surveys were analysed by Chi-square (*χ*^2^) test (replaced by Fisher’s exact test if necessary). Univariable logistic regression was used to determine risk factors for *P. vivax* infection and gametocyte positivity. The crude odds ratios (OR) with their respective 95% confidence intervals (95% CI) were determined considering *P. vivax* infection and gametocyte carriage as the dependent variables. Logistic regression was used for the multivariable analyses and the adjusted ORs (AOR) with 95% CI were also calculated. All variables associated with the outcomes at a significance level of *P* < 0.20 in the univariable analysis were included in the multivariable analysis. Median comparisons for copy number between surveys and between symptomatic and asymptomatic individuals were made using Mann-Whitney test. Differences were considered statistically significant for *P* < 0.05. Association between *P. vivax* parasite density and *P. vivax* gametocyte density was analysed by linear regression.

## Results

### Study population

A total of 4083 participants were included in the two surveys: 1047 people were surveyed only once during the beginning of the rainy season in 2012 (CS1), 1110 only during the 2013 dry season (CS2) and 963 at both time points (Table 1). Overall, 2138 participants were male (52.4%), 507 were aged under 5 years (12.4%), 710 were 5–11 years-old (17.4%), 502 were 12–17 years-old (12.3%), 1974 were 18–59 years-old (48.3%) and 388 were 60 years-old or older (9.5%), with no significant differences regarding age (*χ*^2^ = 2.815, *df* = 4, *P* = 0.589) or gender (*χ*^2^ = 0.119, *df* = 1, *P* = 0.731) between surveys. Besides pre-school (15.0%) and school children (24.4%), 15.4% of participants were adults working in agriculture/fishing, 14.3% worked in an office or pursued high education studies, while 15.6% were house wives and 5.0% retired (Table 1). Ten percent were engaged in other professions or unemployed. The majority of participants had lived in the village for longer than two months (CS1: 93.8%, CS2 97.8%, *χ*^2^ = 40.359, *df* = 1, *P* < 0.0001) and few had travelled outside the village in the month prior to the survey (CS1: 9.4%, CS2: 9.0%, *χ*^2^ = 0.196, *df* = 1, *P* = 0.658) (Table 1).

**Table 1:** Characteristics of study participants

Variable	CS1		CS2		*χ* ^2^	*P*-value
	*n*	%	*n*	%		
Total	2010	100.0	2073	100.0		
Demographic						
Community						
Ipiranga	439	21.8	487	23.5	34.507	< 0.0001
Brasileirinho	1185	59.0	1049	50.6		
Puraquequara	386	19.2	537	25.9		
Gender						
Male	1058	52.6	1080	52.1	0.119	0.731
Female	952	47.4	993	47.9		
Age group						
< 5 yrs	256	12.7	251	12.1	2.815	0.589
≥ 5–11 yrs	361	18.0	349	16.8		
12–17 yrs	253	12.6	249	12.0		
18–59 yrs	957	47.7	1017	49.1		
≥ 60 yrs	181	9.0	207	10.0		
Occupation						
Office worker and higher education student	318	15.8	296	14.3	45.221	< 0.0001
Agriculture/fishing	349	17.4	277	13.4		
Housewife	289	14.4	346	16.7		
Retired	106	5.3	97	4.7		
School children	481	23.9	484	23.4		
Infants and preschool children	312	15.5	300	14.5		
Unemployed/ other	154	7.7	269	13.0		
More than 2 months in the area						
Yes	1866	93.8	2017	97.8	40.359	< 0.0001
No	124	6.2	46	2.2		
Recent travel (last month)						
Yes	188	9.4	186	9.0	0.196	0.658
No	1814	90.6	1883	91.0		
Malaria control						
Use of a bednet						
No use	1002	49.9	1297	62.6	317.571	< 0.0001
< 1 yr	940	46.8	487	23.5		
1–2 yrs	68	3.4	289	13.9		
IRS past 6 months						
Yes	1140	56.7	759	36.6	165.854	< 0.0001
No	869	43.3	1313	63.3		
Fly screen						
Yes	376	18.8	408	19.7	0.565	0.452
No	1627	81.2	1663	80.3		
Malaria morbidity						
Previous episodes						
0	587	29.3	606	29.3		
1–3	656	32.8	614	29.6	6.479	0.091
4–10	455	22.7	487	23.5		
> 10	303	15.1	359	17.3		
Episode in past two weeks						
Yes	24	1.2	21	1.0	0.307	0.580
No	1986	98.8	2052	99.0		
Current symptoms						
Fever^a^	108	5.4	84	4.1	3.974	0.046
Symptoms other than fever^b^	76	3.8	16	0.8	42.857	< 0.0001

*Abbreviations*: CS1, cross-sectional survey 1; CS2, cross-sectional survey 2

^a^> 37.5 °C at visit or past 48 h

^b^Excluding individuals with other symptoms and fever

The majority of participants lived in households that had benefited from vector control activities. In CS1 73.7% (1482/2010) of participants were using LLINs (long-lasting insecticide-treated nets) and/or lived in a house that was treated with indoor spraying of residual insecticides (IRS) in the preceding six months, in CS2 it was 54.5% (1131/2073) of participants (*χ*^2^ = 162.812, *df* = 1, *P *< 0.0001). In CS1, 50.1% (1008/2010) used LLINs, 56.7% (1140/2010) lived in an IRS treated house, and 33.1% (666/2010) of participants had benefited from both interventions. As LLIN distributions and IRS spraying campaigns were conducted in the months prior to CS1, the proportion of people that reported benefitting from either intervention was significantly higher in CS1 than for CS2 (Table 1, *P *< 0.0001). In addition, considering data from both CS1 and CS2, 19.2% (784/4083) of participants lived in households with screened windows.

### *Plasmodium* spp. prevalence

In CS1, 86 of 2010 (4.3%) participants were infected with *P. vivax* as determined by qPCR. Prevalence by qPCR was thus 2.7-fold higher than prevalence by LM (1.6%) (Table 2). Fifty percent (43/86) of all *P. vivax* infected individuals (qPCR) carried *P. vivax* gametocytes when detected by RT-qPCR, whereas 56.3% (18/32) of *P. vivax* infections detected by LM were positive for gametocytes. A modestly lower prevalence of *P. vivax* infections was observed in CS2 with 3.4% (70/2,073) of participants having qPCR-detectable infections, although this difference between surveys did not reach statistical significance, irrespective of considering all participants (Table 2, *χ*^2^ = 2.259, *df* = 1, *P* = 0.133) or only those that were surveyed twice (CS1: 45/963, 4.7% *vs* CS2: 33/963, 3.4%, *χ*^2^ = 1.924, *df* = 1, *P* = 0.165). A comparable number of *P. vivax* infections were gametocyte positive in both surveys (50.0% *vs* 42.9%, *χ*^2^ = 0.791, *df* = 1, *P* = 0.374). Total *P. vivax* parasitemia was comparable in both surveys (median gene copy number/μl 21.8 *vs* 35.0, Mann-Whitney test: *U =* 2643.5, *n*_1_* =* 86, *n*_2_ = 70*, P* = 0.192) but *pvs25* transcript numbers were significantly higher in gametocyte positive samples in CS2 compared to CS1 (median 11.4 *vs* 44.6 transcripts/μl, Mann-Whitney test: *U =* 417.5, *n*_1_* =* 43, * =* *n*_2_ = 30,* P* = 0.011). Overall, *P. falciparum* infections were rare (Table 2): In CS1 only 15 mono *P. falciparum* infections were detected by qPCR (0.8%); in CS2 a single mixed *P. falciparum*/*P. vivax* infection was detected (0.05%, *χ*^2^ = 12.739, *df* = 1, *P* = 0.0004). Among *P. falciparum* positive samples in CS1, 81.8% (9/11) showed presence of gametocytes by light microscopy and 93.3% (14/15) by qPCR. In CS2, the only *P. falciparum* positive sample was also positive for gametocytes by qRT-PCR.

**Table 2:** Prevalence of infection by *P. vivax* (Pv total), *P. vivax* gametocytes (Pv gam), *P. falciparum* (Pf total) and *P. falciparum* gametocytes (Pf gam) as determined by light microscopy and PCR. Microscopy data available only for cross-sectional survey (CS) 1, overall prevalence by PCR determined as *18S* rDNA copies/μl by qPCR, gametocyte prevalence by PCR determined by RT-qPCR (*pvs25* transcripts/μl)

	CS1 Microscopy (*n* = 2010)				CS1 PCR (*n* = 2010)				CS2 PCR (*n* = 2073)			
	*n*	%	Median paras./μl	IQR	*n*	%	Median cop./transcr./μl	IQR	*n*	%	Median cop./transcr./μl	IQR
Pv total	32	1.8	195.0	60.0–555.0	86	4.3	21.8	6.0–246.4	70^a^	3.4	35.0	12.3–273.0
Pv gam	18^b^	1.0	60.0	30.0–60.0	43	2.1	11.4	3.8–85.1	30^a^	1.4	44.6	13.5–175.1
Pf total	11	0.5	240.0	90.0–600.0	15	0.8	151.8	8.1–681.3	1^a^	0.05	5.1	–
Pf gam	9^b^	0.4	150.0	60.0–270.0	14	0.7	434.9	40.0–1272.8	1^a^	0.05	154.8	–

^a^Including one Pf/Pf mixed infection

^b^One slide was negative for asexual blood stages

### Morbidity and univariate risk-factors for *P. vivax* infection

Combining the results of both CS1 and CS2, prevalence of *P. vivax* was about twice as high in Brasileirinho and Puraquequara (4.3%) than in Ipiranga (2.1%, *χ*^2^ = 10.198, *df* = 2, *P* = 0.006) (Additional file 4: Table S4). The proportion of infected individuals was higher among male study participants (4.8%) than among female participants (2.7%) and male participants had a significantly higher risk for infection (OR: 1.81, 95% CI: 1.28–2.58, *χ*^2^ = 12.137, *df* = 1, *P* = 0.0005). Adults (≥ 18 years, prevalence 4.8%) had a higher risk for infection than children (2.4%) (OR: 2.02, 95% CI: 1.40–2.97, *χ*^2^ = 15.369, *df* = 1, *P* = 0.0001). The risk for infection was significantly higher for workers from the agriculture sector than for office workers and individuals pursuing higher education (OR: 1.81, 95% CI: 1.03–3.17, *χ*^2^ = 28.208, *df* = 1, *P* = 0.0001).

Prevalence amongst new residents (4.1%) was only slightly higher than that for individuals that lived in the study area for more than two months (3.8%, OR: 1.08, 95% CI: 0.42–2.32, *χ*^2^ = 0.035, *df* = 1, *P* = 0.852). Individuals with recent travel history showed a lower prevalence (2.4%) than individuals that did not leave the study area (4.0%, OR: 0.60, 95% CI: 0.30–1.18, *χ*^2^ = 2.271, *df* = 1, *P* = 0.132. *Plasmodium vivax* prevalence was higher amongst individuals using LLINs (4.7%, OR: 1.53, 95% CI: 1.11–2.11, *χ*^2^ = 6.796, *df* = 1, *P* = 0.009) and for participants living in IRS-treated houses (4.6%, OR: 1.51, 95% CI: 1.09–2.09, *χ*^2^ = 6.361, *df* = 1, *P* = 0.012) as compared to persons not using either intervention measure (3.1%). The proportion of *P. vivax* infected individuals was lower, but not significant, if the individual´s home had screened windows (3.2%), compared to those individuals living in houses with unscreened windows (4.0%, OR: 0.80, 95% CI: 0.52–1.24, *χ*^2^ = 1.006, *df* = 1, *P* = 0.316).

Study participants that had not experienced malaria episodes during their lifetime showed a lower risk for infection (prevalence 0.8%) than participants with one or more previous malaria infections (5.1%, OR: 7.04, 95% CI: 3.59–15.75, *χ*^2^ = 43.030, *df* = 1, *P* < 0.0001). Furthermore, *P. vivax* prevalence was higher in individuals that experienced a recent malaria episode (7.0%) than in individuals with no recent episode (3.8%, OR: 1.94, 95% CI: 0.77–4.88, Fisher’s exact test *P* = 0.195). Individuals presenting fever the day of the visit or during the past 48 h had a significantly increased risk for infection by *P. vivax* (prevalence 14.1%, OR: 4.77, 95% CI: 3.06–7.43, *χ*^2^ = 57.510, *df* = 1, *P* < 0.0001). Individuals with symptoms other than fever (up to 48 h before visit) had a moderately increased, albeit not-significant, prevalence (5.4%) as compared to asymptomatic individuals (3.63%, OR: 1.55, 95% CI: 0.48–34.83, Fisher’s exact test: *P* = 0.386) (Additional file 4: Table S4).

### Multivariate risk factors for *P. vivax* infection

After adjusting for potential confounders (community, vector control activities and age) gender, occupation, history of malaria and fever remained as independent predictors for *P. vivax* infection. In the multivariate analysis male individuals were at higher risk for infection by *P. vivax* than female individuals (Table 3, AOR: 2.22, 95% CI: 1.41–3.51, *P* = 0.001). Individuals that reported to have had one or more previous malaria episodes in their lives were at much higher risk for *P. vivax* infection than individuals that had no previous infection (Table 3, *P* < 0.001). Another factor strongly associated with *P. vivax* infection was fever (during visit or during the past 48 h) (AOR: 4.66, 95% CI: 2.88–7.53, *P* < 0.001).

**Table 3:** Multivariate predictors of infection by *P. vivax*

	*n*	%	AOR	95% CI	*P*-value
Gender					
Male	2138	4.8	2.22	1.41–3.51	0.001
Female	1945	2.7	–	–	
Occupation					
Office worker and higher education student	614	3.3	–	–	
Agriculture/fishing	626	5.8	1.68	0.96–2.95	0.009
Housewife	635	4.3	2.46	1.28–4.71	
Infants and preschool children	612	2.0	1.47	0.63–3.40	
School children	965	2.5	0.95	0.51–1.78	
Retired	203	3.9	1.12	0.48–2.63	
Unemployed/other	423	6.9	2.13	1.16–3.89	
Number of previous episodes					
0	1193	0.8	–		
1–3	1270	3.9	5.25	2.37–11.65	< 0.001
4–10	942	5.9	7.75	3.46–17.36	
> 10	662	6.2	7.01	2.95–16.64	
Fever^a^					
Yes	192	14.1	4.66	2.88–7.53	< 0.001
No	3891	3.3	–	–	-

^a^> 37.5 °C at visit or past 48 h

### Carriage of *P. vivax* gametocytes

**Fig. 2:**
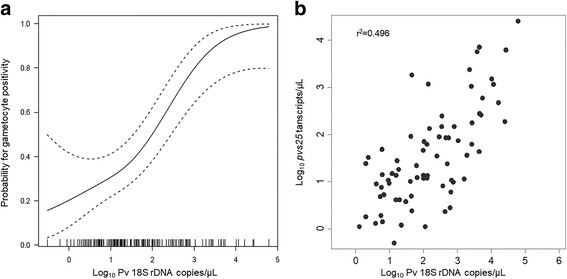
Relation between *P. vivax* parasitaemia and gametocyte carriage and gametocyte density. A generalized additive model was used to describe gametocyte positivity (**a**). *Plasmodium vivax* parasitaemia was determined by qPCR (*18S* rDNA copies/μl) and gametocyte density by RT-qPCR (*pvs25* transcripts/μl) (**b**)

Overall, about half of all *P. vivax* infected individuals (73/156, 46.8%) were positive for gametocytes, detected by RT-qPCR. Both in the univariate (OR: 2.73, 95% CI: 1.85–4.05, *P *< 0.0001) and in the multivariate (AOR: 2.67, 95% CI: 1.90–3.77, *P *< 0.0001) analyses the only risk factor associated with detectable carriage of gametocytes was *P. vivax* parasite density. We modelled the probability for carriage of gametocytes (defined as positivity by RT-qPCR in a 50 μl blood sample) depending on *P. vivax* density using a generalised additive model (Fig. 2a). For low parasite densities (< 10 *18S* rDNA copies/μl) the probability was below 30.0% and increased only slightly. At 100 copies/μl the probability to carry gametocytes was 50.0% and increased dramatically reaching a probability of 90.0% at 10,000 copies/μl. Gametocyte density (measured as *pvs25* transcripts/μl) also increased with *P. vivax* density (*r*^2^ = 0.496, *P *< 0.0001) (Fig. 2b).

**Fig. 3:**
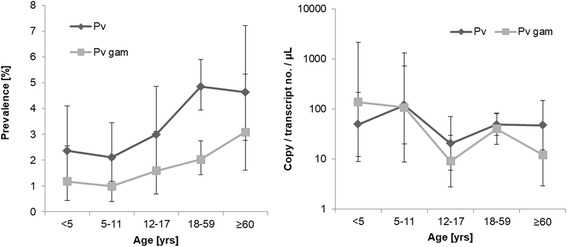
Prevalence of *P. vivax* blood-stages and *P. vivax* gametocytes and parasite densities in different age groups. Geometric mean and 95% confidence interval are shown *Plasmodium vivax* blood-stage (Pv) density was determined by qPCR (*18S* rDNA copies/μl) and gametocyte (Pv gam) density by RT-qPCR (*pvs25* transcripts/μl)

Both prevalence of *P. vivax* blood-stages and prevalence of *P. vivax* gametocytes increased with age. The density of *P. vivax* blood-stages and *pvs25* transcripts was highest in children younger than 12 years (Fig. 3).

### Asymptomatic and submicroscopic infections

**Fig. 4:**
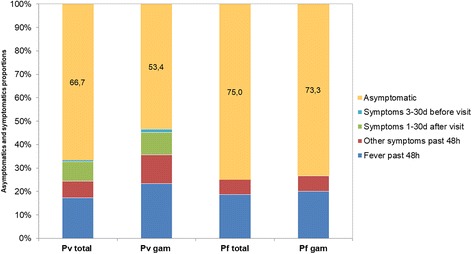
Proportion of asymptomatic infections. Fever and other symptoms as reported on day of visit or up to 48 h before. Symptoms more than 2 days before or after visit according to Information System for Epidemiological Surveillance of Malaria (SIVEP-Malária). *Abbreviations*: Pv total, all *P. vivax* blood stages; Pv gam, *P. vivax* gametocytes; Pf total, all *P. falciparum* blood stages; Pf gam, *P. falciparum* gametocytes

**Fig. 5:**
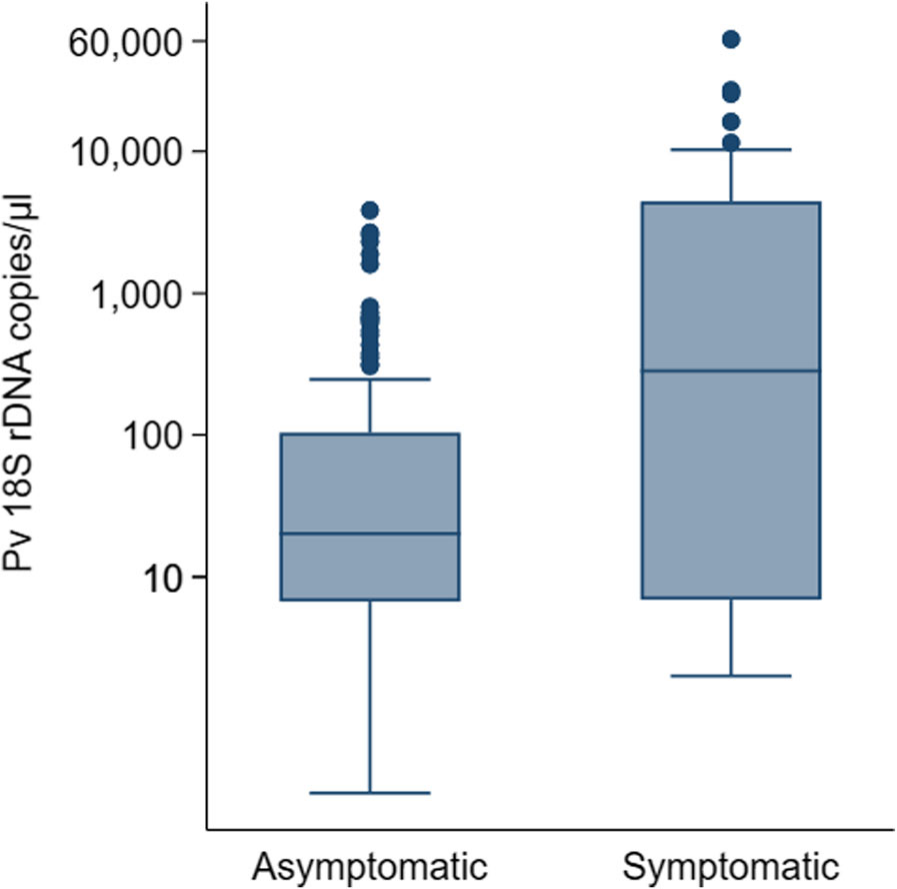
Parasite densities of *P. vivax* infected asymptomatic (*n* = 38) and symptomatic (*n* = 118) individuals as determined by qPCR (*18S* rDNA copies/μl). Box plot showing median and interquartile range. Malaria-related symptoms at the day of visit or up to 48 h before visit were considered

**Fig. 6:**
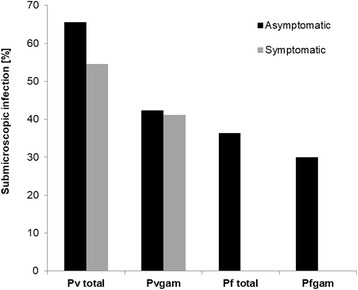
Proportion of submicroscopic infections by *P. vivax* (Pv total), *P. vivax* gametocytes (Pv gam), *P. falciparum* (Pf total) and *P. falciparum* gametocytes (Pf gam) among individuals with or without symptoms (at sample collection or up to 48 h before)

Overall, 82.7% (129/156) of all people infected with *P. vivax* did not report a concurrent febrile illness and 75.0% (117/156) of infected individuals did not report fever or any other symptoms (the day of visit or up to 48 h before) nor did they report with symptoms to a malaria clinic during the 30 days before visit. Of these asymptomatic individuals, 11.1% (13/117) developed symptoms within 30 days after the visit. Thus, 67.3% (104/156) of all *P. vivax* infected individuals were and remained asymptomatic. There was no significant difference between CS1 (56/86) and CS2 (48/70) (*χ*^2^ = 0.2073, *df* = 1, *P* = 0.649). Similarly, 76.7% (56/73) of *P. vivax* gametocyte positive individuals were afebrile and 64.4% (47/73) were asymptomatic. 53.4% (39/73) of individuals carrying *P. vivax* gametocytes were asymptomatic (at visit and past 30 days) and did not develop any symptoms within 30 days after the visit (Fig. 4). Febrile (at visit or past 48 h) *P. vivax* infections were of significantly higher density (median copy numbers/μl: 463.8, IQR: 13.5–5030.4) than afebrile infections (20.8, IQR: 6.7–113.5, Mann-Whitney test: *U =* 985.5, *n*_1_ = 27, *n*_2_ = 129, *P* = 0.0004). Individuals showing any malaria-related symptoms up to the past 48 h also exhibited a significantly higher parasite density (median copy numbers: 282.6, IQR: 6.9–4452.6) as compared to asymptomatic infections (20.3, IQR: 6.7–103.4, *P* = 0.0007, Fig. 5). In CS1, 65.6% (42/64) of asymptomatic and 54.5% (12/22) of symptomatic *P. vivax* infections were submicroscopic (*χ*^2^ = 0.860, *df* = 1, *P* = 0.354) (Fig. 6).

Of the 16 *P. falciparum* infections (including 1 mixed infection), 12 (75.0 %) were asymptomatic (at visit or up to 48 h before). Of the 15 individuals carrying *P. falciparum* gametocytes, 11 (73.3%) were asymptomatic. None of the *P. falciparum* infected individuals developed symptoms within 30 days after the visit (Fig. 4). Four (35.7%) asymptomatic *P. falciparum* infections in CS1 were submicroscopic, while all symptomatic infections were detectable by LM (Fig. 6).

## Discussion

The two cross-sectional surveys conducted in the periphery of Manaus confirmed the low prevalence of malarial infections in the area. This is in accordance with the significant successes recently achieved in Brazil regarding malaria control, whereby from 2000 to 2011 the number of cases has been more than halved [24]. This success has been attributed to classical intervention measures including early recognition and treatment of malaria cases and a high coverage of LLIN distribution [2]. The prevalence observed in CS2 (August-September 2013) was slightly lower than that of CS1 (November-December 2012). Although in the Amazon region malaria transmission usually peaks during the dry period from June to September, year-to-year fluctuations occur due to variation in climatic conditions [6, 17, 25].

Most infections were caused by *P. vivax*; only 14.9% (CS1) and 1.4 % (CS2), respectively, were caused by *P. falciparum*, in accordance with a decreasing trend in *P. falciparum* cases in Brazil since 1989 [25]. In the municipality of Manaus, for instance, *P. falciparum* infections dropped from 40.8% in 1986 to 6.5% in 2003 [7]. In addition to intervention strategies focusing on early diagnosis and treatment, the introduction of artemisinin-based combination therapy in 2006 further enhanced this trend [2]. Early treatment is generally more efficient against *P. falciparum* than *P. vivax* [26], as the appearance of mature gametocytes occurs later during infection. Additionally, the need to ensure radical treatment of *P. vivax* often jeopardizes the adequate management of this species because of compliance issues. As a consequence, in many co-endemic settings successful control has resulted in a shift from *P. falciparum* to *P. vivax* being the most prevalent parasite [27, 28].

Multivariate analysis revealed gender and occupation as key factors associated with infection by *P. vivax*, with male individuals being at higher risk as compared to female. This difference can possibly be explained by different occupation and leisure related behaviour. For instance, one third of adult men were working in the agricultural sector, which was associated with an elevated risk for infection (although not significant). One fifth of adult men had stated to be unemployed or pursue other not classified occupations. That group was significantly associated with infection. Among the different occupation groups housewives were at highest risk for *P. vivax* infection, probably because many houses in the study area include an outside area where activities such as laundry or gardening are performed. Our observations are in agreement with a previously published study conducted in a farming settlement in the South of the Amazon State where an increased risk of infection for individuals working in agriculture or housekeeping occurred [10]. In the univariate model age also represented a risk factor for *P. vivax* infections, showing an elevated risk for adolescents and adults, although this is likely associated with behaviour rather than age. The strongest predictor for *P. vivax* infection was fever. However, one has to keep in mind that in areas with such high proportion of asymptomatically infected individuals, in some cases the fever potentially might be caused by other concurrent (often unrelated) infectious diseases.

We were not able to find a protective effect for LLINs or IRS. Univariate analyses showed instead that both intervention measures were associated with a higher risk for infection by *P. vivax*. This can probably be explained by increased attention and control measures by both health post agents and inhabitants in known transmission hotspots. Whether LLINs and IRS can reduce malaria transmission in South America is not fully clear yet, as the main vector in the region, *An. darlingi*, is to a large extent exophilic, with its main peak of activity occurring at nightfall [29]. In addition, many of the detected *P. vivax* infections might result from reactivated hypnozoites rather than from recent mosquito bites. According to a study carried out in Papua New Guinea, relapses may be the cause of about 80% of *P. vivax* episodes [30]. A recent study from the Brazilian Amazon also suggests a significant contribution of relapses in this region [14].

Amongst individuals positive for *P. vivax*, we identified *P. vivax* blood-stage parasite density as the only risk factor for carrying gametocytes. Other studies also identified (asexual) blood stage density as a major predictor for carriage of gametocytes [31, 32]. Furthermore, we observed a weak correlation between total parasitaemia and gametocyte density. In *P. vivax*, gametocytes appear early in infection and their production continues throughout the infection, thus explaining increased risk for gametocyte carriage and increasing gametocyte densities with increasing *P. vivax* parasitaemia [33].

About half of all *P. vivax* positive individuals were positive for gametocytes. Other reports show higher rates of gametocyte carriers; however, these symptomatic individuals had high parasite densities that correlated with better detection of gametocytes [10, 34, 35]. A study that used large blood volumes for RNA extraction and subsequent RNA extraction achieved a high sensitivity of detecting both asexual and sexual stages [10], while in our study only 50 μl of patient blood was used for RNA extraction. In addition, the proportion of gametocyte positive individuals depends on the sensitivity of the assay detecting parasitaemia of blood-stage parasites - the more sensitive, the lower the parasite densities being detected, and therefore the probability of being positive for (detectable) gametocytes [32]. Gametocyte count by LM was performed on 200 leukocytes in the samples from this study, which may have underestimated the prevalence of gametocytes in infected individuals. The sensitivity of gametocyte detection could be improved counting more microscopic fields.

Up to three quarters of all *P. vivax* infected individuals appeared to be free of symptoms on the day of blood sampling. The majority of them also remained free of symptoms for the following 30 days, as previously reported [10, 15]. We were able to demonstrate a high proportion of asymptomatic infections in a low transmission setting, as previously described in the Americas [15, 36].

Among the individuals positive for *P. vivax* gametocytes, the proportion of asymptomatic individuals was smaller. This can be explained by the lower chance to detect gametocytes in low blood-stage parasite densities, such as observed in asymptomatic individuals. Despite having lower parasitemia, and therefore lower gametocytemia, asymptomatic infections had been shown to be able to infect mosquitoes [37] and hence, might be an important reservoir, especially for seasonal outbreaks [26]. A study in southern Amazon State estimated that 54.4% of all parasite biomass belonged to asymptomatic infected individuals, accounting for 56.6% of all infections [14]. As asymptomatic carriers do not seek healthcare facilities, they do not receive treatment and therefore might remain infective over long periods. Furthermore, a large proportion of asymptomatic infections is submicroscopic and would therefore by standard diagnostic methods remain undetected. Overall, more than half of *P. vivax* infections (62.8%) were submicroscopic, as opposed to only 26.7 % of *P. falciparum* infections. Two recent reviews reported similar proportions of subpatent (69.5% [38] and 67.0% [39]) *P. vivax* infections and substantially lower proportions of subpatent *P. falciparum* infections in various studies from different continents. In Brazil only LM positive infections are treated, thus the malaria subpatent infections remain untreated.

This work is the first large-scale survey assessing the infectious reservoir, and therefore the malaria transmission potential, with molecular methods in the peri-urban area of Manaus. We identified a large proportion of asymptomatic and submicroscopic malaria infections implicating that these might play an important role in malaria transmission in the study area. These infections are not only a burden to the local population, but also responsible for infections in (malaria-naïve) visitors [6]. Classical intervention measures are not effective against subpatent infections and new control measures will therefore be needed to achieve elimination. Although PCR is not suitable for routine diagnostics, as it is expensive and technically challenging, molecular data as those arising from our study can provide important information on the infectious reservoir, for instance for mathematical models [39]. A clearer understanding of the dynamics of the infectious reservoir over time is needed and longitudinal studies will be critical in order to assess the duration of asymptomatic infections and their likely role in sustaining transmission in the area.

## Conclusions

This study shows a substantial proportion of asymptomatic and submicroscopic *P. vivax* infections in the studied area. The majority of gametocyte carriers did not present symptoms, thus indicating that asymptomatic infections might significantly contribute to malaria transmission in this region. Therefore, efficient control and malaria elimination tools need to include approaches also targeting the asymptomatic reservoir. Longitudinal studies are needed in these locations to analyze the incidence of malaria infection and the risk factors for *P. vivax* gametocyte carriage.

## Additional files

Additional file 1: **Table S1.** Primers and probes used for amplification of *18S* rRNA, *pvs25* and *pfs25*, using qPCR or RT-qPCR assays including reaction efficiency and detection limits. (DOCX 16 kb)

Additional file 2: **Table S2.** Reaction mixes for amplification of *18S* rRNA, *pvs25* and *pfs25*, using qPCR or RT-qPCR. The table describes the reagents, concentrations and template volume used in the qPCR and RT-qPCR assays. (DOCX 13 kb)

Additional file 3: **Table S3.** Conditions for amplification of *18S* rRNA, *pvs25* and *pfs25*, using qPCR or RT-qPCR assays. (DOCX 14 kb)

Additional file 4: **Table S4.** Univariate analysis for predictors of *P. vivax* infection. (DOCX 22 kb)

## Abbreviations

AOR: Adjusted OR
CI 95%: 95% confidence onterval
CONEP: Brazilian National Committee of Ethics
CS: Cross-sectional
EDTA: Ethylenediaminetetraacetic acid
FMT-HVD: Fundação de Medicina Tropical Dr Heitor Vieira Dourado
IQR: Interquartile range
IRS: Indoor residual spraying
LLINs: Long-lasting insecticide-treated nets
LM: Light microscopy
OR: Odds ratio
PBS: Phosphate buffered saline
PCR: Polymerase chain reaction
Pf gam: *P. falciparum* gametocytes
Pf: *P. falciparum* blood-stage
Pv gam: *P. vivax* gametocytes
Pv: *P. vivax* blood-stage
qPCR: Quantitative PCR
RBC: Red blood cells
RT-qPCR: Quantitative reverse transcription PCR
SIVEP-Malaria: System for Epidemiological Surveillance of Malaria
