# Supplementary material for: High proportions of asymptomatic and submicroscopic Plasmodium vivax infections in a peri-urban area of low transmission in the Brazilian Amazon
Source: Parasit Vectors. 2018 Mar 20;11:194. doi: 10.1186/s13071-018-2787-7 (PMC5859403; doi:10.1186/s13071-018-2787-7)
Supplement: Supplementary file 1 — Table S1. Primers and probes used for amplification of 18S rRNA, pvs25 and pfs25, using qPCR or RT-qPCR assays including reaction efficiency and detection limits. (DOCX 16 kb) [file 13071_2018_2787_MOESM1_ESM.docx]

Additional file 1: Primers and probes used for amplification of *18S rRNA genes,* *pvs25* and *pfs25*, using qPCR or RT-qPCR.

| **Assay** | **Primers/Probes** | **PCR Efficiency** | **Assay detection limit** | **Ref.** |
| --- | --- | --- | --- | --- |
| Qmal  (*18S rRNA* gene) | Fw-TTA GAT TGC TTC CTT CAG TRC CTT ATG | 90.6% | 1 copy/uL |  |
|  | Rev-GT TGA GTC AAA TTA AGC CGC AA |  |  | 18 |
|  | Probe: **FAM** – TCA ATT CTT TTA ACT TTC TCG CTT GCG CGA – **BHQ1** |  |  |  |
| *P.falciparum*-specific  (*18S rRNA* gene) | Fw-TAT TGC TTT TGA GAG GTT TTG TTA CTT TG | 90.5% | 3 copies/uL | 22 |
|  | Rev-TATTCCATGCTGTAGTATTCAAACACAA |  |  | 23 |
|  | Probe: **FAM** – ACG GGT AGT CAT GAT TGA GTT – **MGB** – **NFQ** |  |  | 22 |
| *P. vivax*-specific  (*18S rRNA* gene) | Fw- GCT TTG TAA TTG GAA TGA TGG GAA T | 92.2% | 1 copy/uL | 22 |
|  | Rev- ATG CGC ACA AAG TCG ATA CGA AG |  |  |  |
|  | Probe: **VIC** – AGC AAC GCT TCT AGC TTA – **MGB** – **NFQ** |  |  |  |
| *P. falciparum* gametocyte *(Pfs25* transcript) | Fw- GAA ATC CCG TTT CAT ACG CTT G | 94.5% | 1 copy/uL | 18 |
|  | Rev- AGT TTT AAC AGG ATT GCT TGT ATC TAA |  |  |  |
|  | Probe: **HEX** – TGT AAG AAT GTA ACT TGT GGT AAC GGT- **BHQ1** |  |  |  |
| *P. vivax* gametocyte  *(Pvs25* transcript) | Fw- ACA CTT GTG TGC TTG ATG TAT GTC | 96.2% | 0,5 copy/uL | 18 |
|  | Rev- ACT TTG CCA ATA GCA CAT GAG CAA |  |  |  |
|  | Probe: **FAM** –TGC ATT GTT GAG TAC CTC TCG GAA- **BHQ1** |  |  |  |
