# Supplementary material for: High proportions of asymptomatic and submicroscopic Plasmodium vivax infections in a peri-urban area of low transmission in the Brazilian Amazon
Source: Parasit Vectors. 2018 Mar 20;11:194. doi: 10.1186/s13071-018-2787-7 (PMC5859403; doi:10.1186/s13071-018-2787-7)
Supplement: Supplementary file 2 — Table S2. Reaction mixes for amplification of 18S rRNA, pvs25 and pfs25, using qPCR or RT-qPCR. The table describes the reagents, concentrations and template volume used in the qPCR and RT-qPCR assays. (DOCX 13 kb) [file 13071_2018_2787_MOESM2_ESM.docx]

Additional file 2. Reaction mixes for amplification of *18S rRNA genes*, *pvs25* and *pfs25*, using qPCR or RT-qPCR.

| **Assay** | **Reagents** | **Concentration** | **Total Volume Reaction** | **DNA/RNA Volume** |
| --- | --- | --- | --- | --- |
| Qmal  (*18S rRNA* gene) | TaqMan GeneExpression MasterMix (Applied Biosystems)  Primers Fw+Rev  Probe | 1x  833nM  417nM | 12 µL | 4µL |
| *P.falciparum*-specific  (*18S rRNA* gene) | TaqMan Universal PCR MasterMix (Applied Biosystems)  Primers Fw+Rev  Probe | 1x  600nM  250nM | 12 µL | 4µL |
| *P. vivax*-specific  (*18S rRNA* gene) | TaqMan GeneExpression MasterMix (Applied Biosystems)  Primers Fw+Rev  Probe | 1x  600nM  417nM | 12 µL | 4µL |
| *P. falciparum* gametocyte  *(Pfs25* transcript) | TaqMan 1-Step MasterMix (Applied Biosystems)  Primers Fw+Rev  Probe  TaqMan Reverse Transcriptase Enzyme | 1x  833nM  417nM  1x | 12 µL | 4µL |
| *P. vivax* gametocyte  *(Pvs25* transcript) | TaqMan 1-Step MasterMix (Applied Biosystems)  Primers Fw+Rev  Probe  TaqMan Reverse Transcriptase Enzyme | 1x  833nM  417nM  1x | 12 µL | 4µL |
