# Supplementary material for: High proportions of asymptomatic and submicroscopic Plasmodium vivax infections in a peri-urban area of low transmission in the Brazilian Amazon
Source: Parasit Vectors. 2018 Mar 20;11:194. doi: 10.1186/s13071-018-2787-7 (PMC5859403; doi:10.1186/s13071-018-2787-7)
Supplement: Supplementary file 3 — Table S3. Conditions for amplification of 18S rRNA, pvs25 and pfs25, using qPCR or RT-qPCR assays. (DOCX 14 kb) [file 13071_2018_2787_MOESM3_ESM.docx]

Additional file 3: Conditions for amplification of *18S rRNA genes*, *pvs25* and *pfs25*, using qPCR or RT-qPCR

| Assay | Stage | Step | Temperature | Time |
| --- | --- | --- | --- | --- |
| Qmal (*18S rRNA gene*),  *P. falciparum* specific (*18S rRNA gene*),  *P. vivax* specific (*18S rRNA gene*) | Holding | Pre-Incubation | 50°C | 2 minutes |
|  | Holding | Activation of Taq polymerase | 95°C | 10 minutes |
|  | Cycling (45x) | Denature | 95°C | 15 seconds |
|  |  | Anneal/Extend | 58°C | 1 minute |
| *P. falciparum* gametocyte  (*Pfs25* transcript),  *P. vivax* gametocyte  (*Pvs25 transcript*) | Holding | Reverse transcription | 48°C | 15 minutes |
|  | Holding | Activation of Taq polymerase | 95°C | 10 minutes |
|  | Cycling (45x) | Denature | 95°C | 15 seconds |
|  |  | Anneal/Extend | 58°C | 1 minute |
