# Supplementary material for: High proportions of asymptomatic and submicroscopic Plasmodium vivax infections in a peri-urban area of low transmission in the Brazilian Amazon
Source: Parasit Vectors. 2018 Mar 20;11:194. doi: 10.1186/s13071-018-2787-7 (PMC5859403; doi:10.1186/s13071-018-2787-7)
Supplement: Supplementary file 4 — Table S4. Univariate analysis for predictors of P. vivax infection. (DOCX 22 kb) [file 13071_2018_2787_MOESM4_ESM.docx]

Additional file 4: Univariate predictors of infection by *P. vivax*.

|  | n | % | OR | CI (95%) | P-value |
| --- | --- | --- | --- | --- | --- |
|  |  |  |  |  |  |
| **Total** | 4,083 | 3.8 |  |  |  |
| ***Demographic*** |  |  |  |  |  |
| **Community** |  |  |  |  |  |
| Ipiranga | 926 | 2.1 |  |  |  |
| Brasileirinho | 2,234 | 4.3 | 2.17 | 1.31-3.77 | 0.006 |
| Puraquequara | 923 | 4.3 | 2.16 | 1.21-3.98 |  |
| **Gender** |  |  |  |  |  |
| Male | 2,138 | 4.8 | 1.81 | 1.28-2.58 | <0.001 |
| Female | 1,945 | 2.7 |  |  |  |
| **Age group** |  |  |  |  |  |
| <5 yrs | 507 | 2.4 |  |  |  |
| ≥5 - 11 yrs | 710 | 2.1 | 0.89 | 0.39-2.10 | 0.003 |
| 12 - 17 yrs | 502 | 3.0 | 1.27 | 0.55-3.01 |  |
| 18-59 yrs | 1,974 | 4.9 | 2.11 | 1.14-4.26 |  |
| ≥60 yrs | 388 | 4.6 | 2.01 | 0.90-4.62 |  |
| **Occupation** |  |  |  |  |  |
| Office worker & higher education student | 614 | 3.3 |  |  |  |
| Agriculture/ pisciculture | 626 | 5.8 | 1.81 | 1.01-3.34 | <0.001 |
| House wife | 635 | 4.3 | 1.31 | 0.70-2.51 |  |
| Infants and preschool children | 612 | 2.0 | 0.59 | 0.26-1.29 |  |
| School children | 965 | 2.5 | 0.76 | 0.40-1.46 |  |
| Retired | 203 | 3.9 | 1.22 | 0.46-2.94 |  |
| Unemployed/ other | 423 | 6.9 | 2.19 | 1.18-4.14 |  |
| ***Malaria Control*** |  |  |  |  |  |
| **Regular use of LLINs** |  |  |  |  |  |
| Yes | 1,784 | 4.7 | 1.53 | 1.09-2.14 | 0.01 |
| No | 2,299 | 3.1 |  |  |  |
| **IRS past 6 months** |  |  |  |  |  |
| Yes | 1,899 | 4.6 | 1.51 | 1.08-2.12 | 0.01 |
| No | 2,182 | 3.1 |  |  |  |
| **Fly screen** |  |  |  |  |  |
| Yes | 784 | 3.2 | 0.80 | 0.50-1.25 | 0.32 |
| No | 3,290 | 4.0 |  |  |  |
| ***Malaria Morbidity*** |  |  |  |  |  |
| **Previous episodes** |  |  |  |  |  |
| 0 | 1,193 | 0.8 |  |  |  |
| 1 - 3 | 1,270 | 3.9 | 5.28 | 2.55-12.27 | <0.001 |
| 4 - 10 | 942 | 5.9 | 8.32 | 4.05-19.21 |  |
| >10 | 662 | 6.2 | 8.64 | 4.10-20.34 |  |
| **Fever (>37.5ºC)^b^** |  |  |  |  |  |
| Yes | 192 | 14.1 | 4.77 | 2.94-7.51 | <0.001 |
| No | 3,891 | 3.3 |  |  |  |
| **Symptoms other than fever^c^** |  |  |  |  |  |
| Yes | 92 | 5.4 | 1.70 | 0.53-4.23 | 0.23^a^ |
| No | 3,799 | 3.3 |  |  |  |
|  |  |  |  |  |  |
| ^a^ Fisher's exact test |  |  |  |  |  |
| ^b^ Fever at visit or past 48 h |  |  |  |  |  |
| ^c^ at visit, excluding individuals with fever |  |  |  |  |  |
|  |  |  |  |  |  |
